# A 115,000-year-old expedient bone technology at Lingjing, Henan, China

**DOI:** 10.1371/journal.pone.0250156

**Published:** 2021-05-06

**Authors:** Luc Doyon, Zhanyang Li, Hua Wang, Lila Geis, Francesco d’Errico

**Affiliations:** 1 Institute of Cultural Heritage, Shandong University, Qingdao, China; 2 CNRS UMR5199 –PACEA, Université de Bordeaux, France; 3 SSF Centre for Early Sapiens Behavior (SapienCe), University of Bergen, Bergen, Norway; Universita degli Studi di Ferrara, ITALY

## Abstract

Activities attested since at least 2.6 Myr, such as stone knapping, marrow extraction, and woodworking may have allowed early hominins to recognize the technological potential of discarded skeletal remains and equipped them with a transferable skillset fit for the marginal modification and utilization of bone flakes. Identifying precisely when and where expedient bone tools were used in prehistory nonetheless remains a challenging task owing to the multiple natural and anthropogenic processes that can mimic deliberately knapped bones. Here, we compare a large sample of the faunal remains from Lingjing, a 115 ka-old site from China which has yielded important hominin remains and rich faunal and lithic assemblages, with bone fragments produced by experimentally fracturing *Equus caballus* long bones. Our results provide a set of qualitative and quantitative criteria that can help zooarchaeologists and bone technologists distinguish faunal remains with intentional flake removal scars from those resulting from carcass processing activities. Experimental data shows marrow extraction seldom generates diaphyseal fragments bearing more than six flake scars arranged contiguously or in interspersed series. Long bone fragments presenting such characteristics can, therefore, be interpreted as being purposefully knapped to be used as expediency tools. The identification, based on the above experimental criteria, of 56 bone tools in the Lingjing faunal assemblage is consistent with the smaller size of the lithics found in the same layer. The continuity gradient observed in the size of lithics and knapped bones suggests the latter were used for tasks in which the former were less or not effective.

## Introduction

Owing to their ubiquity in the archaeological record since 3.3 Myr (million years ago) [1, 2], stone tools have attracted much attention in studies of the technological changes associated with the evolution of members of our lineage. Despite use wear evidence for woodworking [3, 4] and bone cutting [5], the latter likely resulting from butchery and carcass processing activities, it remains unclear how and when lithic and organic technologies integrated the technical system of our ancestors and how they co-evolved. The origin and early developments of organic technologies remain difficult to apprehend because of their perishable nature. Pinpointing when osseous artefacts were incorporated in past technological system is nonetheless decisive in palaeoanthropological research because it identifies a significant shift in the way prehistoric human groups conceived faunal resources at their disposal. Specifically, it signals when animal skeletal element utility expanded to include the manufacture of implements in addition to their primary role for consumption, fat use or fuel. Earliest examples of osseous tools include bone digging implements from Southern Africa, an innovation attributed to *Australopithecus robustus* living in this region some 2.0–1.5 Myr ago as well as bone fragments bearing evidence of intentional flaking, battering and abrasion from Olduvai Beds I and II, East Africa, likely used by early members of our genus, *Homo*, in hide-working, butchery, digging, knapping, and hunting activities between ~1.8–1.0 Myr [6–10]. In the Southeast Asian Pacific Islands, shell scrapers were found at Trinil, Java [11], in a formation linked to *Homo erectus* occupation some 450 kyr (thousand years ago). In Europe and the Levant, many Lower Palaeolithic antler, bone, and ivory tools were reported, yet most of them have been repeatedly called into questions [for a review see 12, 13, and references therein]. An indubitable tool type, however, consists of Acheulean bone handaxes. These tools are documented in Africa, at Olduvai Bed II, 1.7–1.15 Myr [7], and at Konso, Ethiopia, in a context dated to ~1.4 Myr [14], in numerous sites dated between ~500–250 kyr from the Levant at Revadim Quarry [15], Central Europe at Vértesszőlős and Bilzingsleben [16, 17], and Southern Europe at Torre in Pietra, La Polledrara, Fontana Ranuccio and Castel di Guido [18–22], as well as at the Bashiyi Quarry, Chongqing, China, in a context dated to ~170 kyr [23].

Archaeologists usually make a distinction between two main bone tool categories: formal tools, i.e., faunal remains formally shaped into specific tool type with manufacturing techniques specific to osseous materials, such as grinding, gouging, scraping, notching, incising, etc., and expedient tools, i.e., bone fragments bearing little or no modifications and that were used as such [24, 25]. It is probable that activities attested since at least 2.6 Myr such as stone knapping, bone fracturing for marrow extraction [26, 27], and woodworking [3, 4] have allowed early hominins to recognize the technological potential of discarded carcass processing remains and equipped them with a transferable skillset fit for the manufacture and utilization of osseous material. Through trials and errors, Palaeolithic hominins would have been able to observe how bone responded to static and dynamic loadings, and embody this knowledge for immediate or future use [sensu 28].

Identifying precisely when expedient tool use became commonplace in our evolutionary history remains a challenging task. Perhaps the most documented amongst this tool category are bone hammers and retouchers, i.e., knapping implements respectively used to remove flakes from lithic cores and to retouch the edges of stone tools. The earliest known instances of these tool types date back to 2.1–1.5 Myr at Olduvai Gorge, Africa [7], to MIS18 (Marine Isotopic Stage) at Gesher Benot Ya’aqov in the Levant [29], MIS13 at Boxgrove in Europe [30] and MIS5 at Lingjing in East Asia [31, 32]. From MIS9, bone retouchers become an integral part of the cultural repertoire of Neanderthals [33–38] and reach during MIS5 a high degree of standardization [36, 39, 40]. Possible expedient tool types also include long bone shaft fragments with one or more edges modified by blows that generated flake scars present on the cortical and/or the medullar surface of the bone. In Europe, growing evidence for this technology appears during MIS9 at Gran Dolina, Spain [41], Schöningen, Germany [42], and in Italy at Castel di Guido [22], Bucobello [43], La Polledera di Cecanibbio [44] and Rebibbia-Casal de’ Pazzi [19]. They were likewise found at Saint-Marcel cave, France, in a context attributed to the MIS4 or the beginning of MIS3 [45]. In East Asia, similar tools were reported at Donggutuo, from a formation dated to 1.2–1.0 Myr [46] as well as at Panxian Dadong in a context dated between 250–130 ka, although the latter were produced on rhinoceros’ teeth [47]. Other instances of expedient bone tools from this region are reported in the literature, e.g., at Xujiayao [48], Zhoukoudian Upper Cave [49], and Yonggul Cave [50], but would require further assessment with modern methods to verify their chronology and the anthropogenic nature of the modifications. It has been proposed that these tools were used for cutting soft animal tissues, vegetal fibers, or as wedges for splitting wood, antler and bone [42, 51–56].

Despite this expanding data set, we are still lacking diagnostic criteria to distinguish faunal remains with flake scars that were intentionally modified for technological purposes from those resulting from carcass processing activities (see Research background). Our aim here is to contribute to the establishment of such criteria. The need for this study arose when analyzing the faunal assemblage excavated at Lingjing, layer 11, an archaeological context dated to 125–105 kyr [57] that has also yielded important archaic human remains [58]. During the 2005–2015 excavation campaigns, one of us (ZL) isolated a number of faunal fragments bearing flake removal scars on both their cortical and medullar surfaces, and interpreted some of them as probable bone tools based on putative use wear recorded on some edges [59]. In 2016, two of us (LD, FD) were invited to re-examine these objects and reappraise a larger sample of faunal remains from the same context bearing flake scars and other modifications to test the hypothesis that they were used as tools. This led to the identification of the earliest known bone and antlers fragments used as retouchers and soft hammer from China [31]. Our research on the flaked specimens takes into account several lines of evidence: 1) a critical review of the site formation process; 2) a thorough quantification of the size and location of the flake removal scars on the putative bone tools; 3) a comparison with a selection of bone fragments isolated during the 2005–2015 excavations (*n* = 127), 4) a randomly selected sample of diaphyseal fragments (*n* = 100) coming from the same layer and recovered during the same excavation seasons (2005–2015), 5) an analysis of the entire faunal assemblage recovered from layer 11 during the 2017 campaign (*n* = 1260); 6) an experimental breakage of large mammals long bones aimed to quantify flake scars resulting from this activity. Our results suggest at least 56 faunal fragments can be interpreted as expedient bone tools, which expands the behavioural realm of the hominins who visited the Lingjing site during the Middle to Late Pleistocene transition.

## Research background

The technological use of carcass processing by-products by prehistoric hominins has been suggested and documented for more than a century. In the early 1900s, Dr. Henri-Martin experimented with the fracturing of horse long bones for marrow extraction and highlighted that some of the resulting bone fragments would have been fit for hide working or for transforming other kinds of material. Comparisons between his experimental results and the faunal remains from the Mousterian layers at La Quina, France, allowed him to suggest criteria to identify expedient osseous tools, such as the presence of use wear in the form of an unevenly distributed polish and worn edges smoothed by friction [60]. Likewise, Raymond Dart hypothesized that instead of knapped lithics, Makapansgat *Australopithecus prometheus* used bone, tooth, and horn as hunting weapons [61]. Despite his interpretation being later attributed to non-anthropogenic, taphonomic processes [62], Dart’s work sparked an interest for studies aimed to document the natural and anthropogenic processes responsible for the modification of faunal remains. We have since gained a clearer understanding of the multiple agents that can cause the post-mortem flaking, cracking, and fragmentation of osseous remains, including gnawing, chewing, fracturing, and digestion by mammal and reptile predators, carnivores, rodents, herbivores, or birds [12, 63–81], fracturing by hominins for marrow and bone grease exploitation [82–93], trampling, root etching, weathering, exposure to heat and cold, sediment pressure, deposition in alkaline environment [62, 63, 94–105], etc.

When osseous technology is concerned, and leaving aside bone retouchers, which have received much attention [e.g., 31–34, 36–39, 106–108 and references therein], the identification of expedient bone tools still heavily relies on the presence of use wear associated with flaking scars on both archaeological [42, 53–55] and experimental specimens [56], accidental fracture and crushing of the working edges and surfaces [51, 52, 109], or a combination of these factors [6, 7, 9, 110]. Faunal remains bearing only flake scars, however, have been somewhat overlooked. In recent years, their description was mainly concerned with flakes produced in the context of osseous tool blank extraction [111, 112]. One noticeable exception remains the experimental work on elephant bones and the archaeological comparison with the assemblage from Olduvai Gorge, Tanzania, where the number of flake removals, their location and dimensions were systematically recorded [7]. In the present paper, we extend the approach proposed by these authors with the aim to distinguish between intentionally modified expedient osseous tools and marrow exploitation by-products from the Lingjing site, Henan, China.

## Archaeological context

**Fig 1:**
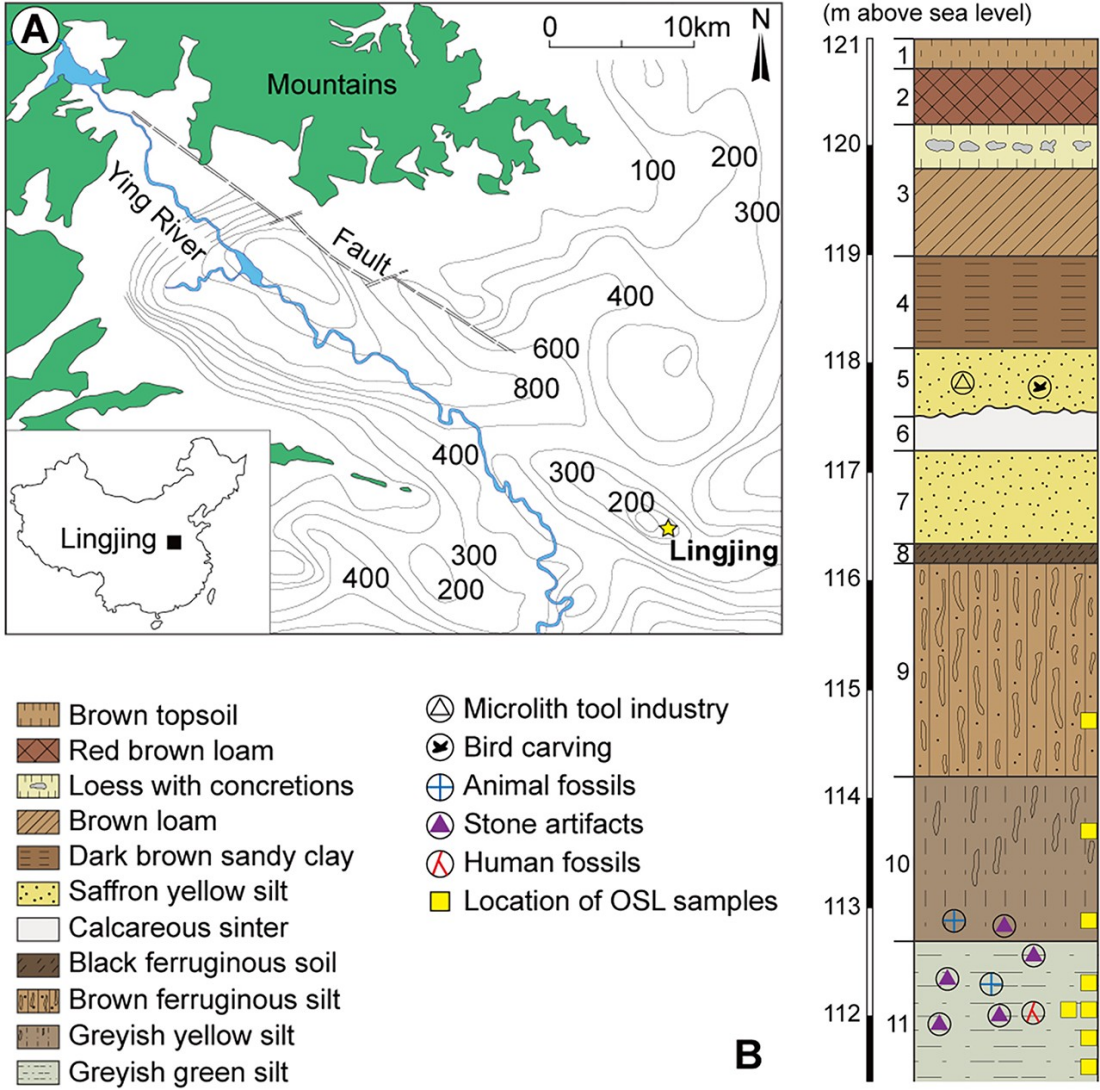
Location of the Lingjing site, and schematic representation of the stratigraphy. Reprinted from [31] under a CC BY license, original copyright 2018.

The Lingjing site was identified in 1965 when microcores and microblades lithic technologies as well as mammalian fossils were collected on the surface of a field [113, 114] in the northeast Xuchang County, Henan Province (34˚ 04’ 08.6” N, 113˚ 40’ 47.5” E, 117masl). The site, a water-lain deposit, is located in a transitional area between the eastern foothills of Songshan Mountains and the Huang-Huai Plain, on the southern fringes of the North China Plain, some 120km south of the Yellow River (Fig 1). Within the vicinity of the site, a number of small ponds that lacked outflows appeared from water welling up an underground river into depressions found along the trailing edge of the Yinghe River. The Lingjing site corresponds to one such pond [58]. Layer 11, which is the focus of the present study, was formed from the horizontal accumulation of sediments originating from one of these springs, as attested by the fine-grained sediment with no evidence of substantial horizontal flow dynamics [115]. A water cistern was built over the opening of the spring present in the southern portion of the site in 1958 [116].

From 2005 to 2017, a *c*. 550m^2^ area was excavated by one of us (ZL, in collaboration with Dr. Hao Li, Institute of Vertebrate Paleontology and Paleoanthropology, Beijing, in 2017) at a depth averaging *c*. 9m. Excavations have been halted since 2018 owing to the construction of an Archaeological Site Museum above the deposit, an infrastructure project which aims to put on display the human fossils and archaeological remains recovered at this locality [see 117 for a definition of this type of Museum]. During the excavations, eleven geological layers were identified and three archaeological horizons, each encompassing one or more geological layers. The uppermost archaeological horizon includes layers 1 to 4; the middle one corresponds to layer 5; and the lowermost comprise layers 10 and 11. Layers 1–4 are Holocene in age and were identified over the entire excavated surface. The archaeological remains recovered from these layers were exclusively found along the northern limit of the investigated area, and only consist of a few isolated, fine pottery sherds, none of which could be refitted to one another. Decors on their outer surface suggest a cultural attribution to a period spanning from the Yangshao Neolithic to the Shang-Zhou Bronze Age (~6.5–2.5 kyr). Layer 5 and the spoil heap left by well diggers in 1958 were identified solely in the southern portion of the site. This layer and the sediments originating from it yielded a rich microcore and microblade industry made of high-quality black chert [118–120], a small amount of quartz tools, some very fragile, thick, crude, simple-shaped pottery sherds with plain surfaces [121], burnt and unburnt faunal remains, charcoals, ostrich egg shell fragments, including one transformed into a perforated pendant, and the oldest sculpture discovered in China, a bird figurine carved from a mammalian long bone fragment that had likely been heated in an anaerobic environment prior to shaping the artwork [116]. The ^14^C dating of burnt bones, charcoals and charred residues recovered on the pottery sherds suggests three human occupations spanning from the Tardiglacial to the Pleistocene-Holocene transition, i.e., a first occupation between ~13.8–13.0 kyr by Tardiglacial hunter-gatherers bearing microlithic technologies who made the bird figurine, and two human occupations by ceramics users between ~11–10 kyr and ~9.6–8.7 kyr respectively. Layers 6 to 9 were identified over the whole excavated area. They were entirely sterile and represent a *c*. 4.5m hiatus between the Tardiglacial human occupations from layer 5 above and the early Late Pleistocene archaeological horizon below.

Layers 10 and 11 were deposited during the early Late Pleistocene. Two OSL samples collected at the base and in the upper half of layer 10 were dated to ~102 ± 2 and ~96 ± 6 kyr respectively. The five OSL samples from layer 11 yielded ages spanning from ~105 kyr at the top to ~125 kyr at the bottom of the layer [57]. These ages correspond to the early MIS5, i.e., MIS5e to MIS5d, and to the last interglacial paleosol S1 in the Chinese Loess Plateau sequence. In 2007 and 2014, 45 fragments of archaic human crania were recovered *in situ* in layer 11. Aside from three isolated pieces, all fragments were refitted into two individual crania [58], named Xuchang (XUC) 1 and 2 after the County in which the site is located. Morphological analysis of the crania identifies a mosaic of anatomical traits that remains undocumented to this day in the Old World. They exhibit ancestral features reminiscent of early Middle Pleistocene eastern Eurasians, others derived and shared by archaic and modern Late Pleistocene individuals as well as a combination of traits at the midoccipital area and the temporal labyrinths usually observed only in Neanderthal populations. This peculiar mix suggests complex intra- and interregional population dynamics between western and eastern Eurasian hominins prior and during the Middle to Late Pleistocene transition. It has been suggested these two individuals could be Denisovans [122] although DNA and proteomic analyses are still missing to test this hypothesis. From a palaeopathological perspective, both XUC1 and XUC2 present external auditory exostoses, i.e., a dense bony growth protruding in the external auditory canal that implies conductive hearing loss [123].

The rich lithic assemblage from Lingjing, layers 10 and 11, amounts to more than 15,400 remains. Quartz and quartzite are the two predominant raw materials used for the manufacture of tools. Alterations of the cortex still present on lithic artefacts, estimation of the original size of the river pebbles selected for knapping, and outcrops survey of the Yinghe River suggest the prehistoric occupants at Lingjing exploited raw material found within 10km from the site [124]. Differences in selected raw material are documented between layer 10 and 11. While the percentage of quartz artefacts declines from layer 11 to 10, this latter layer attests for a diversification of raw material, with a notable increase of quartzite, sandstone and basalt [125]. All products and by-products of the operational sequence are represented in the lithic assemblage. The reduction sequence is mainly oriented towards the detachment of flakes and production of chunks that are later retouched and shaped into tools. A fifth of the cores are of discoidal type; the remaining cores correspond to expedient debitage following a number of knapping strategies [for a distinction between formal and expedient cores, see 126]. This pattern indicates some degree of behavioural flexibility and a proximal, problem-oriented response to satisfy needs requiring the use of lacerating edges [124]. The shaping of blanks into tools is predominantly performed by free-hand hard hammer percussion (≈75%), although organic soft hammer percussion and pressure retouch were also documented on >20% of the implements [124]. The lithic toolkit primarily includes scrapers, notches, denticulates, borers, and points. A few burins and backed pieces were also identified. Rare instances of heavy-duty tools such as choppers and spheroids were documented [124, 125]. Use wear analysis suggests some tools were used at the site [127]. Lithic refitting attempts indicate stone tools were not submitted to significant horizontal or vertical post-depositional disturbances [125].

Ideal post-depositional and fossilization conditions allowed to recover from layer 10 and 11 a rich faunal assemblage surpassing 50,000 remains [128–130]. The carnivore guild is diverse and includes, in decreasing order, *Pachycrocuta* cf. *sinensis*, *Panthera* cf. *tigris*, *Ursus* sp., *Vulpes* sp., *Canis* cf. *lupus*, and *Meles* sp. Dozens of coprolites from medium-sized carnivores, likely hyena, were recovered at the site [131, 132]. The herbivore guild is dominated by equids, i.e., *Equus hemionus* and *Equus przewalskii*, and bovids, i.e., *Bos primigenius*. In decreasing order, the herbivores also include *Coelodonta antiquitanis*, *Sus lyddekeri*, *Cervus elaphus*, *Procapra przewalskii*, *Cervus* (*Sika*) sp.. Other taxa, e.g., *Palaeoloxodon* sp., *Dicerorhinus mercki*, *Hydropotes pleistocenica*, *Elaphurus davidianus*, and *Sinomegaceros ordosianus*, are present but in very small proportions, i.e., usually less than five elements per species [130]. Modifications by carnivore, e.g., pits and scores as well as surface etching owing to digestion, were seldom observed on the faunal remains (<1%), which suggests they played a limited role in the accumulation of the assemblage [31, 32, 133–136]. The main anthropogenic modifications recorded on the faunal remains consist of cut marks generated during butchery activities, and percussion scars likely resulting from the breaking of diaphysis to extract bone marrow. The skeletal element profiles dominated by body parts with lower nutritional values, the mortality patterns of the main prey species, i.e., equids and bovids, represented exclusively by prime-adult individuals, and bone surface modifications demonstrate the importance of the Lingjing site in subsistence activities, namely for the hunting of prey and carcass processing [133–136].

A few dozen bone retouchers were identified, which were grouped into two strategies [31, 32]. The first strategy encompasses 85% of the specimens, and consists of selecting bone fragments and using them as such for a single retouching event to sharpen the dull edges of stone tools likely used in butchery activities. The second strategy involves selecting weathered cervid’s metapodials, marginally modifying them by flaking to produce an elongated tool with improved ergonomic, transportability and efficiency, and intensively, and recurrently use them for retouching stone tools. Alongside the bone retouchers, a single dear antler bears traces of use as soft hammer [31]. Surface modifications observed on a few faunal fragments and their experimental replications suggest some skeletal remains were used in passive and active pressure flaking activities [32], which support Li’s et al. [124] contention for an independent origin of pressure flaking in China *c*.115 ka, i.e., 40,000 years prior to the earliest occurrence of similar behaviour in Southern Africa [137–139].

The use of bone in knapping activities is not restricted to the manufacture and maintenance of stone tools. Numerous bovids and equids metapodia display a combination of alterations, i.e., crushing and flaking on the distal condyles as well as evidence of fresh bending fractures resulting in the sectioning of the distal epiphysis and the main shaft. These modifications have been interpreted as evidence for the intentional selection and use of bovids and equids metapodia for knapping mammal long bones in an attempt to extract the marrow it contains [130]. Interestingly, this behaviour has been also reported at the Schöningen 13 II-4 site, i.e., the Spear Horizon [109, 140–142], which is dated to *c*. 300 ka BP.

Perhaps the most unexpected find from layer 11 consists in the identification of two fragments of medium to large-size mammal rib bearing respectively 10 and 13 sub-parallel engraved lines. Microscopic analysis indicates these lines were made when the fragments were already weathered, therefore rejecting the hypothesis that they could represent butchery cut marks. Analysis of red residues identified in and between the lines engraved on one specimen demonstrates the presence of red hematite, interpreted as evidence of smearing ochre over the pattern to make it more visible [143].

Formation processes of layers 10 and 11 were investigated with magnetic susceptibility, sedimentology, X-ray fluorescence (XRF) and X-ray diffraction (XRD) as well as the orientation and plunge of lithic artefacts. Results suggest a slow deposition rate with limited to low energy flow across the site. Layer 11 likely formed in a relatively stable, close, oxygen-poor environment; the deposition of layer 10 occurred at a time when the local water table was subjected to more frequent rises and falls [115]. These conclusions are supported by the taphonomic analysis of the faunal assemblage. The faunal remains from layer 10 were mainly affected by weathering; those from layer 11 show significantly more elements with surfaces covered with concretions and altered by root-etching [32]. Palaeoenvironmental reconstruction from pollens recovered in hyena coprolites suggests a grassland-dominated vegetation with a mosaic of scattered, mixed forests [131, 132]. This environment, combined with the presence of an active water spring surely attracted both animals and humans throughout the early Late Pleistocene, as attested by the uninterrupted vertical distribution of lithic and faunal remains from the lower half of layer 10 to the bottom of layer 11 [115].

## Material and methods

### Archaeological remains

The faunal assemblage from Lingjing, layer 10 and 11, is curated at the Henan Provincial Institute for Cultural Relics and Archaeology, Zhengzhou, China. No permits were required for the described study, which complied with all relevant regulations. From 2005 to 2016, excavation methods at the site involved removing the sediments with curved-tipped trowels, 3D-plotting faunal and lithic remains with maximum length greater than 2.5cm, and sieving sediments through a 2mm mesh. Both lithic and faunal remains were cleaned using soft brushes under running water. When present, concretions were not removed from the faunal remains. In 2017, the same protocol was implemented, although piece plotting was also performed for fragments measuring 1–2.5cm in length. The material considered in the present study comes exclusively from layer 11 and amounts to 1,487 faunal remains. It includes (1) a sub-sample of 127 bone fragments isolated by one of us (ZL) during the 2005–2015 excavations, and analyzed by two of us (LD, FD) in 2016. The specimens comprised in this sample, henceforth PBT (Potential Bone Tools), bear features, i.e., flake scars, polish, impacts, morphology, that have attracted the attention of the excavator and convinced him they could have been expedient tools; (2) a randomly-selected sub-sample of 100 long bone fragments from the same excavation years, analyzed by two of us (LD, FD) in 2016. This sample, henceforth RCS (Restricted Control Sample), was selected with the purpose of verifying whether PBT or some specimens within PBT stand out in some respects when compared to RCS or simply represent an extreme in variation of the modifications present in the assemblage. Aside from ten specimens bearing remnants of trabeculae, all others are shaft fragments; (3) the entire faunal assemblage yielded by the 2017 excavation of layer 11 leaving aside cranial elements, i.e., 1,260 bone fragments, analyzed by one of us (LD) in 2018. Comprising all post-cranial faunal remains recovered that year, including 1–2,5cm-long fragments, this assemblage composed mainly of diaphyseal fragments (>85%), henceforth CCS (Complete Control Sample), is particularly appropriate for comparison with bone fragments stemming from our experiments since we recovered all bone fragments, including those smaller than 2.5cm.

Each specimen was first examined with a magnifying glass with incident light. Anthropogenic modifications were distinguished from natural ones based on published criteria, with a particular attention on the natural and anthropogenic processes that could produce flaking scars on faunal remains, e.g., trampling, carnivore alterations, marrow extraction [12, 63, 65, 66, 70, 78, 85, 95–98, 105, 144–154], etc. When identifying the cause for specific bone surface modifications proved difficult, microscopic observations were conducted using a Leica Wild M3C stereomicroscope equipped with a Nikon CoolPix 900 digital camera at magnifications ranging from 4–40x. Selected specimens were photographed with a Canon PowerShot 100 and a Nikon D300 AF equipped with a Micro Nikkor 60 mm f/2.8D lens cameras.

Morphometric data, i.e., maximum length, width, thickness, and cortical thickness of the bone fragments, were collected using a digital caliper. The following variables were recorded for specimens with flake scars: number of scars, their location (cortical or medullar surface, distal or proximal, one side or both sides), arrangement (isolated, contiguous, interspersed), and the breadth of each flake scar longer than 0.5mm. We included in the contiguous flake scar category adjacent and overlapping removals. Interspersed series of flake scars refer to two or more sets of contiguous flake scars separated by an unmodified portion of the diaphyseal fragment edge.

### Experimental program

In an attempt to establish if marrow extraction activities could produce a flaking pattern akin to that observed on the faunal remains from Lingjing, we implemented an experimental protocol that aimed to fracture large mammal long bones to expose the marrow. We selected six long bones from an adult *Equus caballus*: two humeri, two tibiae, one femur, and one radius. The choice of taxon was motivated by the fact that equids constitute the majority of the herbivore guild at Lingjing. The horse was killed in Eastern Europe six days prior to the experiment, and kept in a refrigerated room at 4–5˚C before being shipped to the Nouvelle-Aquitaine region by refrigerated truck the day before the experiment. The meat was removed overnight by a professional butcher using modern tools and the bones were received with scraps of meat and connective tissues still attached. They were fractured without previously removing the periosteum and adhering soft tissues.

The fracturing experiment took place on the University of Bordeaux campus. A 6m^2^ woven plastic tarp was placed on a grassy ground to ease the recovery of bone fragments. Two trained experimenters broke the long bones: a 35–40 years-old male, with eight years of experience (henceforth, Series 1), and a 60–65 years-old male, with ~35 years of experience (henceforth, Series 2). Their aim was to produce longitudinal diaphyseal fragments while exposing the marrow. They were free to choose the hitting points and change them throughout the experiment. The bone was resting on a limestone anvil and was secured with one hand holding an epiphysis. With a 1.85kg beach pebble serving as hammerstone in the other hand, the experimenters produced a series of blows on the diaphysis. Two techniques were used. For Series 1, the experimenter started by hitting multiple times a single point on the metaphysis, i.e., the transitional zone at which the diaphysis and epiphysis meet. When cracks started to appear, he did the same on the opposite metaphysis to expand the fracture from the other end of the bone and, then, exposed the marrow by hitting the diaphysis on its mid-section. This procedure was applied on one specimen of each skeletal element. For Series 2, the experimenter hit the diaphysis with a series of rapid, successive blows along the diaphysis from one metaphysis to the opposite. If the marrow was not exposed following the first series, he turned the bone to hit it on a second surface. This procedure was applied to one humerus and one tibia. Although the blows applied to the tibia produced longitudinal fractures, the periosteum prevented the opening of the diaphysis, which was achieved by hitting the bone directly on the anvil. The batting technique was not used in our experiment. This choice was motivated by the fact that no blocks suitable for this fracturing method were found at the site.

Throughout the experiment, notes were taken by a third participant (LG) on recording sheets where the anterior, posterior, medial, lateral, proximal and distal aspects of each element were illustrated. The information recorded includes the location of the percussion, the number of blows as well as any qualitative observations made by the experimenters in the process. Photographs and video recordings were done with a Canon PowerShot G7 X Mark II camera. After the breakage of each bone, all bone fragments and epiphyses were collected in a single bag associated with an identification code indicating the date of the experiment, the element, and the series’ number. Broken bones were cleaned separately to avoid loss of small fragments and/or identification codes at the *Laboratoire de Préparation des faunes* (UMR5199 PACEA, University of Bordeaux). This experimental reference collection, curated at UMR5199 PACEA, is available for studying and teaching purposes.

The broken bones were separated into four categories: epiphysis, diaphyseal fragments, flakes, and splinters. Diaphyseal fragments correspond to large bone pieces preserving at least 10% of the shaft circumference, where both cortical and medullar surfaces are present and that can be refitted, at least mentally, to other fragments. Flakes refer to medium-sized remains, usually larger than 2cm, that were detached either from the cortical or the medullar surface. The shaft circumference cannot be estimated from this category and, unless bearing clear anatomical features or a bulb of percussion and/or a morphology matching a flake scar on a diaphyseal fragment, they prove difficult to refit with other pieces. Splinters consists of small bone pieces, usually less than 2cm in length. They outnumber any other categories and sometime preserve small remains of cortical and/or medullar surfaces indicating their original position within the diaphyseal section. They are too small to allow their refitting to any other pieces. Epiphyses were not considered in the present study. Morphometric and qualitative data collection on the cleaned diaphyseal fragments, flakes, and splinters followed the procedure described for the archaeological samples. In addition, for flakes and splinters, we established their original position relative to the cortical thickness (cortical, medullar surface, or unknown), and recorded the presence of the percussion bulb.

Statistical tests and data representation were performed in R-CRAN [155]. The maximum lengths recorded on diaphyseal fragments from each sample were compared with the Kruskal-Wallis non-parametric test because the values were not normally distributed, therefore preventing the use of an ANOVA. Thickness values were normally distributed and differences between samples were tested with Student’s *t*-test. An ANOVA complemented with a pairwise comparison based on Tukey HDS was applied to test for significance differences in the number of flake scars recorded on bone fragments from each sample.

## Results

### Experimental data

**Fig 2:**
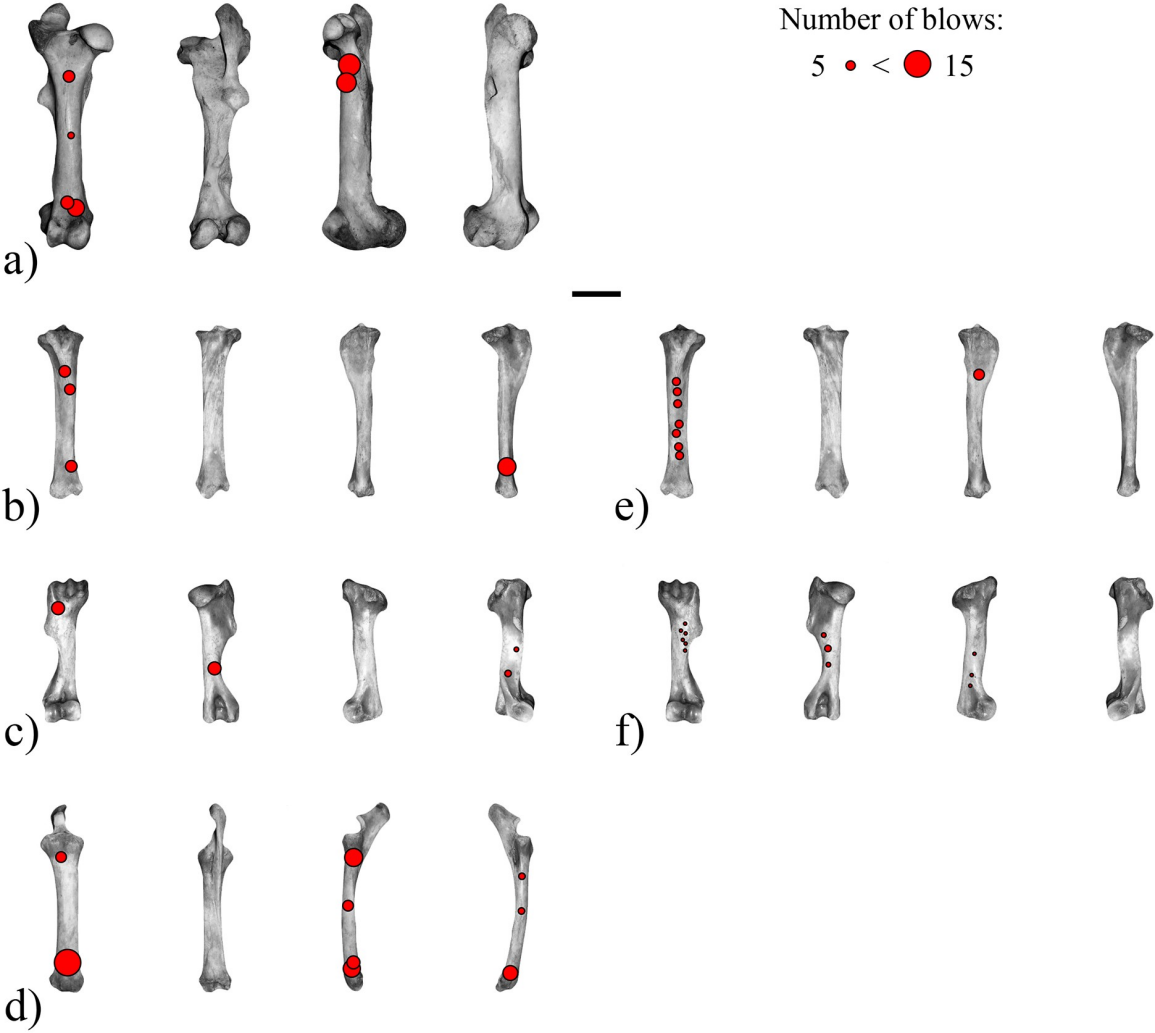
Location and frequency of the blows produced with a hammerstone during the experimental marrow extraction on *Equus caballus* long bones (for each element, from left to right, anterior, posterior, medial, and lateral aspect). (a-d) Series 1: (a) femur, (b) tibia, (c) humerus, (d) radius. (e-f) Series 2: (e) tibia, (f) humerus. Scale = 10 cm.

On average, ~38 blows were necessary to expose the medullar cavity (Fig 2). This average is reduced to 25 blows (minimum: 16 blows for the humerus from Series 2; maximum: 34 blows for the tibia from Series 2) when the femur and radius from Series 1 are not considered, which respectively required 62 and 65 blows to access the marrow. Compared to fracturing experiments done on cattle long bones [e.g., 86, 90, 93], more blows were required to expose the medullar cavity, which can be explained by the more invasive and dense spongy bone present in horse’s long bones. The final blow performed on the tibia from Series 2 resulted in the high fragmentation of its diaphysis. Remains from the fracturing of Series 2’s tibia are left out of the presentation of the experimental results; they are, however, included in the comparison with the archaeological samples (see below).

Both methods used to break the bones produced comparable number of fragments, flakes and, to a lesser extent, splinters (Table 1). Percussion bulbs are present on 35.71% of the flakes and on 8.66% of splinters (μ = 11.73%). When the original position of flakes and splinters within the diaphyseal section is examined, both categories show an average of 14.23% specimens detached from the medullar surface. Flakes are almost six times (5.75 to 1) more likely to be detached from the cortical surface of the bone than from the medullar one. Likewise, splinters are twice (2.17 to 1) more likely to detach from the cortical surface. This difference is mainly due to the high proportion of splinters of unknown origins (55.12%). When size is considered, and despite a greater dispersion around the mean, Series 1 has consistently produced fragments with lengths on average three times longer than their widths. The humerus’ fragments from Series 2, on the other hand, are on average twice as long as they are large. This result suggests it would have been possible for Palaeolithic hominins to apply both knapping methods in the event they wanted to produce elongated blanks while simultaneously exploiting bone marrow.

**Table 1:** Proportion of specimens bearing a percussion bulb, relative frequencies of the origin of fragments, flakes, and splinters, and morphometric data of the remains produced during the experimental marrow extraction on *Equus caballus* long bones by experimenter.

Series	Element	Category	*n*	% w/ perc. bulb*	Flakes & Splinters’ Origin					Dimensions (in mm)		
					% Cortical	% Medular	% Both	% Unknown		Maximum Length	Maximum Width	Maximum Thickness
1	Humerus	Fragment	3						μ	102.06	33.84	16.79
									σ	45.97	14.01	14.89
		Flake	7	2 (28.57%)	3 (42.86%)	2 (28.57%)	2 (28.57%)	0 (0.00%)	μ	31.38	13.66	3.98
									σ	12.86	3.07	0.91
		Splinter	18	0 (0.00%)	5 (27.78%)	3 (16.67%)	1 (5.56%)	9 (50.00%)	μ	11.06	5.58	2.14
									σ	5.95	2.81	1.13
	Radius	Fragment	7						μ	102.15	33.05	17.05
									σ	26.71	9.19	7.34
		Flake	8	2 (25.00%)	3 (37.50%)	1 (12.50%)	4 (50.00)%	0 (0.00%)	μ	43.25	16.95	9.00
									σ	8.90	6.17	4.29
		Splinter	34	6 (17.65%)	10 (29.41%)	4 (11.76%)	0 (0.00%)	20 (58.82%)	μ	14.10	5.95	2.22
									σ	5.60	2.90	1.25
	Femur	Fragment	8						μ	97.82	37.99	24.22
									σ	34.38	13.36	14.44
		Flake	3	1 (33.33%)	3 (100.00%)	0 (0.00%)	0 (0.00%)	0 (0.00%)	μ	21.59	16.90	12.45
									σ	1.26	5.26	7.87
		Splinter	28	0 (0.00%)	4 (14.29%)	7 (25.00%)	3 (10.71%)	14 (50.00%)	μ	19.09	7.47	3.25
									σ	10.43	2.84	2.26
	Tibia	Fragment	2						μ	190.78	59.48	35.10
									σ	NA	NA	NA
		Flake	3	2 (66.67%)	1 (33.33%)	1 (33.33%)	1 (33.33%)	0 (0.00%)	μ	56.61	20.14	8.55
									σ	21.17	7.30	2.49
		Splinter	33	4 (12.12%)	9 (27.27%)	2 (6.06%)	3 (9.09%)	19 (57.58%)	μ	21.81	7.22	2.90
									σ	15.34	3.94	2.07
2	Humerus	Fragment	4						μ	133.30	64.20	42.66
									σ	23.01	10.72	16.98
		Flake	7	3 (42.86%)	3 (42.86%)	0 (0.00%)	3 (42.86%)	1 (14.29%)	μ	34.01	18.38	5.96
									σ	10.75	4.67	3.24
		Splinter	14	1 (7.14%)	2 (14.29%)	2 (14.29%)	2 (14.29%)	8 (57.14%)	μ	12.93	7.08	3.19
									σ	4.92	3.21	1.90
	Tibia	Fragment	9						μ	66.04	28.76	15.67
									σ	19.41	8.77	5.19
		Flake	21	2 (9.52%)	5 (23.81%)	9 (42.86%)	4 (19.05%)	3 (14.29%)	μ	37.48	14.17	33.60
									σ	8.91	3.10	117.21
		Splinter	108	2 (1.85%)	13 (12.04%)	15 (13.89%)	1 (0.93%)	79 (73.15%)	μ	13.16	17.47	2.46
									σ	5.87	118.38	1.39

* percentage of each remain category bearing a percussion bulb.

Half of the diaphyseal fragments (18 out of 33) bear flake scars (Table 2). In almost 90% of the cases, fragments with clear flake removal scars also bear indubitable impact scars, i.e., small depressions or crushing of the compacta produced by the protrusion of the object used to hit the bone shaft, and eventually break it and expose the medullar cavity to access the marrow. When the location of the flake scars is considered (Table 3), they are more often present on the cortical (48.75%) than on the medullar surface (30.00%) or on both (21.25%). They also occur mainly on the distal and/or proximal edges of the fragments (51.25%) than on the sides (28.25%). This pattern is characteristic of comminuted fractures resulting from high-impact and high-energy compressive trauma on the bone diaphysis [156]. It has been observed in other fracturing experiment, e.g., the breaking of elephant long bone through a variety of techniques [7]. Flake removals on the medullar surface of the bone are systematically associated with percussion notches presenting, on the medullary view, multiple superimposed conchoidal scars [sometimes called overlapping notches or percussion notches with inner conchoidal scars 86, 92]. Repeated impacts on a small area of the cortical surface accelerate the detachment of flakes in, or near, the corresponding area on the medullar surface.

**Table 2:** Summary of the morphometric data for the samples considered in the present study and comparison with specimens bearing flake scars by sample.

			All faunal remains					Only faunal remains with flake removal scars					
Origin	Sample	Year	*n*		Maximum Length	Maximum Width	Maximum Thickness	*n*	% w/ impact scars*		Maximum Length	Maximum Width	Maximum Thickness
Archaeological	PBT	2005–2015	127	μ	80.44	29.47	13.73	77	5%	μ	86.64	32.09	14.85
				σ	39.64	13.81	8.51			σ	41.44	15.22	8.78
	RCS	2005–2015	100	μ	56.66	21.32	11.82	44	0%	μ	61.95	24.45	12.27
				σ	25.15	10.36	6.31			σ	27.95	8.21	4.97
	CCS	2017	1260	μ	45.76	21.03	11.82	17	0%	μ	86.92	33.96	17.46
				σ	30.37	13.03	9.07			σ	37.52	13.35	7.87
Experimental	Series 1	2020	154	μ	31.94	11.91	5.28	10	90%	μ	125.11	40.92	21.62
				σ	35.94	11.96	7.33			σ	48.41	14.23	10.70
	Series 2	2020	163	μ	23.04	17.96	8.40	8	88%	μ	92.37	41.83	24.95
				σ	24.00	96.63	42.95			σ	43.70	19.56	13.26

PBT = Potential bone tools; RCS = Restricted control sample; CCS = Complete control sample (see Materials and methods for details on the sampling).

* Percentage of fragments with co-occurrence of flake removal scars and impact scars.

**Table 3:** Relative proportion for the location and arrangement of flake scars by sample considered in the present study.

Sample	Location of flake removal scars	Cortical			Medullar			Bifacial		
		Isol.	Cont.	Int. Ser.	Isol.	Cont.	Int. Ser.	Isol.	Cont.	Int. Ser.
**Exp. Series 1 (*n* = 10)**	**4 sides**									
	**Prox AND/OR Dist**	2 (20.0%)	1 (10.0%)		1 (10.0%)					
	**Prox AND/OR Dist AND Lat**			1 (10.0%)				3 (30.0%)		
	**Lat ONLY 1**		1 (10.0%)							
	**Lat ONLY 2**	1 (10.0%)								
**Exp. Series 2 (*n* = 8)**	**4 sides**									
	**Prox AND/OR Dist**		2 (25.0%)		2 (25.0%)				1 (12.5%)	
	**Prox AND/OR Dist AND Lat**									
	**Lat ONLY 1**	1 (12.5%)			2 (25.0%)					
	**Lat ONLY 2**									
**PBT (*n* = 77)**	**4 sides**								1 (1.3%)	4 (5.2%)
	**Prox AND/OR Dist**		7 (9.1%)					3 (3.9%)	2 (2.6%)	
	**Prox AND/OR Dist AND Lat**		4 (5.2%)	2 (2.6%)			1 (1.3%)	1 (1.3%)	11 (14.3%)	15 (19.5%)
	**Lat ONLY 1**	2 (2.6%)	11 (14.3%)	1 (1.3%)	2 (2.6%)	2 (2.6%)			4 (5.2%)	
	**Lat ONLY 2**			2 (2.6%)			1 (1.3%)			1 (1.3%)
**RCS (*n* = 44)**	**4 sides**									
	**Prox AND/OR Dist**	4 (9.5%)	2 (4.8%)					4 (9.5%)		1 (2.4%)
	**Prox AND/OR Dist AND Lat**	2 (4.8%)	3 (7.1%)	2 (4.8%)		1 (2.4%)		2 (4.8%)		4 (9.5%)
	**Lat ONLY 1**	4 (9.5%)	1 (2.4%)		7 (15.9%)	4 (9.5%)		1 (2.4%)	1 (2.4%)	
	**Lat ONLY 2**								1 (2.4%)	
**CCS (*n* = 17)**	**4 sides**									
	**Prox AND/OR Dist**	2 (11.8%)	4 (23.5%)			1 (5.9%)				
	**Prox AND/OR Dist AND Lat**		2 (11.8%)	1 (5.9%)						2 (11.8%)
	**Lat ONLY 1**	1 (5.9%)			2 (11.8%)					
	**Lat ONLY 2**						1 (5.9%)			1 (5.9%)

Isol. = Isolated; Cont. = Contiguous; Int. Ser. = Interspersed series.

Prox = Proximal end; Dist = Distal end; Lat = Lateral edge

PBT = Potential bone tools; RCS = Restricted control sample; CCS = Complete control sample (see Materials and methods for details on the sampling).

When the arrangement of flake scars is considered, almost two thirds of them (66.25%) are isolated. It is rare to count more than two isolated flake scars on a single diaphyseal specimen. Only two diaphyseal fragments deviate from this rule and present four and seven flake scars; they respectively come from the fracturing of the femur and radius, i.e., the two bones from Series 1 that required the greatest number of blows to open the medullar cavity. Our experiment suggests marrow exploitation can produce contiguous flake scars 28.75% of the time. Contiguous flake scars vary between two to three per fragments. Only one diaphyseal fragment from Series 2’s tibia bears six contiguous flake scars at its distal end, i.e., five on the cortical and one on the medullar surface. Interspersed series of flake scars were observed only on one specimen (5%), i.e., the radius from Series 1, which presents three contiguous flake scars at its distal end and a single flake scar on its side, near the distal end.

### Archaeological data

The faunal remains from Lingjing generally present an excellent state of preservation. The main taphonomic modification recorded on the faunal assemblage from layer 11 is root etching (Table 4); this damage is observed on 36.31% of the overall remains, and in somewhat greater proportions when considering only diaphyseal fragments bearing flake scars (44.93%). No traces of abrasion or trampling were observed on the specimen included in this study. Modification caused by carnivores are rare (1.08%, or 16 specimens out of 1487). The most common anthropogenic modification consists of butchery cut marks (18.51%). A few impact scars reflecting deliberate bone fracture were also identified (2.38%). With the exception of four specimens from the PBT, none of the other diaphyseal fragments with flake scars bear impact scars that could be interpreted as resulting from bone fracturing activities. The degree of polish of the surface of faunal remains is quite variable and substantially higher on the PBT specimens, whether they bear flake scars or not, compared to the other two archaeological samples, i.e., RCS and CCS (Table 4). This pattern can be in part explained by the fact that the specimens comprised in this sample were set aside specifically because of the features they presented, i.e., the co-occurrence of polish and flake scars or peculiar morphology. When considering only RCS and CCS, polish surfaces and edges are more often observed, albeit in low intensity, on specimens from the former sample than on the latter regardless if they bear flake scars or not (Table 4). This observation suggests that polish, in and of itself, is a poor indicator to differentiate whether a bone fragment was intentionally transformed into a tool by marginal knapping and used at Lingjing. In some instance, polish may be explained by natural processes although further investigation is required to establish their precise origin.

**Table 4:** Morphometric data on the compacta thickness and relative frequencies for natural and anthropogenic alterations recorded on the archaeological samples from Lingjing, layer 11, based on the presence or absence of flake scars on the specimens.

										Polish											
			Compacta Thickness (mm)			Carnivore				Cortical surface				Medullar surface				Edges			
	Sample	*n*	μ	σ	Root etching	Scoring	Diges-tion	Marrow extrac-tion	Cut marks	Low	Medium	High	None	Low	Medium	High	None	Low	Medium	High	None
With flake removal scars	PBT	77	9.36	4.00	33 (42.9%)	3 (3.9%)	1 (1.3%)	4 (5.2%)	26 (33.8%)	11 (14.3%)	42 (54.5%)	17 (22.1%)	7 (9.1%)	25 (32.5%)	31 (40.3%)	4 (5.2%)	17 (22.1%)	20 (26.0%)	36 (46.8%)	8 (10.4%)	13 (16.9%)
	RCS	44	9.18	5.42	19 (43.2%)	0 (0.0)%	0 (0.0%)	0 (0.0%)	18 (8.2%)	17 (38.6%)	5 (11.4%)	0 (0.0%)	22 (50.0%)	7 (15.9%)	2 (4.5%)	0 (0.0%)	35 (79.5%)	5 (11.4%)	2 (4.5%)	0 (0.0%)	37 (84.1%)
	CCS	17	8.61	3.58	10 (58.8%)	0 (0.0%)	0 (0.0%)	0 (0.0%)	1 (5.9%)	1 (5.9%)	0 (0.0%)	1 (5.9%)	15 (88.2%)	0 (0.0%)	0 (0.0%)	1 (5.9%)	16 (94.1%)	0 (0.0%)	1 (5.9%)	0 (0.0%)	16 (94.1%)
Without flake removal scars	PBT	50	6.73	2.84	15 (30.0%)	5 (10.0%)	4 (8.0%)	3 (6.0%)	12 (24.0%)	13 (26.0%)	12 (24.0%)	5 (10.0%)	20 (40.0%)	8 (16.0%)	5 (10.0%)	0 (0.0%)	37 (74.0%)	6 (12.0%)	6 (12.0%)	0 (0.0%)	38 (76.0%)
	RCS	56	7.02	4.03	18 (32.1%)	0 (0.0%)	0 (0.0%)	1 (1.8%)	8 (14.3%)	24 (42.9%)	1 (1.8%)	0 (0.0%)	31 (55.4%)	7 (12.5%)	0 (0.0%)	0 (0.0%)	49 (87.5%)	5 (8.9%)	0 (0.0%)	0 (0.0%)	51 (91.1%)
	CCS	1243	5.79	3.03	445 (35.8%)	4 (0.3%)	5 (0.4%)	27 (2.2%)	221 (17.8%)	80 (6.4%)	7 (0.6%)	0 (0.0%)	1156 (93.0%)	48 (3.9%)	2 (0.2%)	0 (0.0%)	1192 (95.9%)	15 (1.2%)	11 (0.9%)	0 (0.0%)	1217 (97.9%)

PBT = Potential bone tools; RCS = Restricted control sample; CCS = Complete control sample (see Materials and methods for details on the sampling).

**Fig 3:**
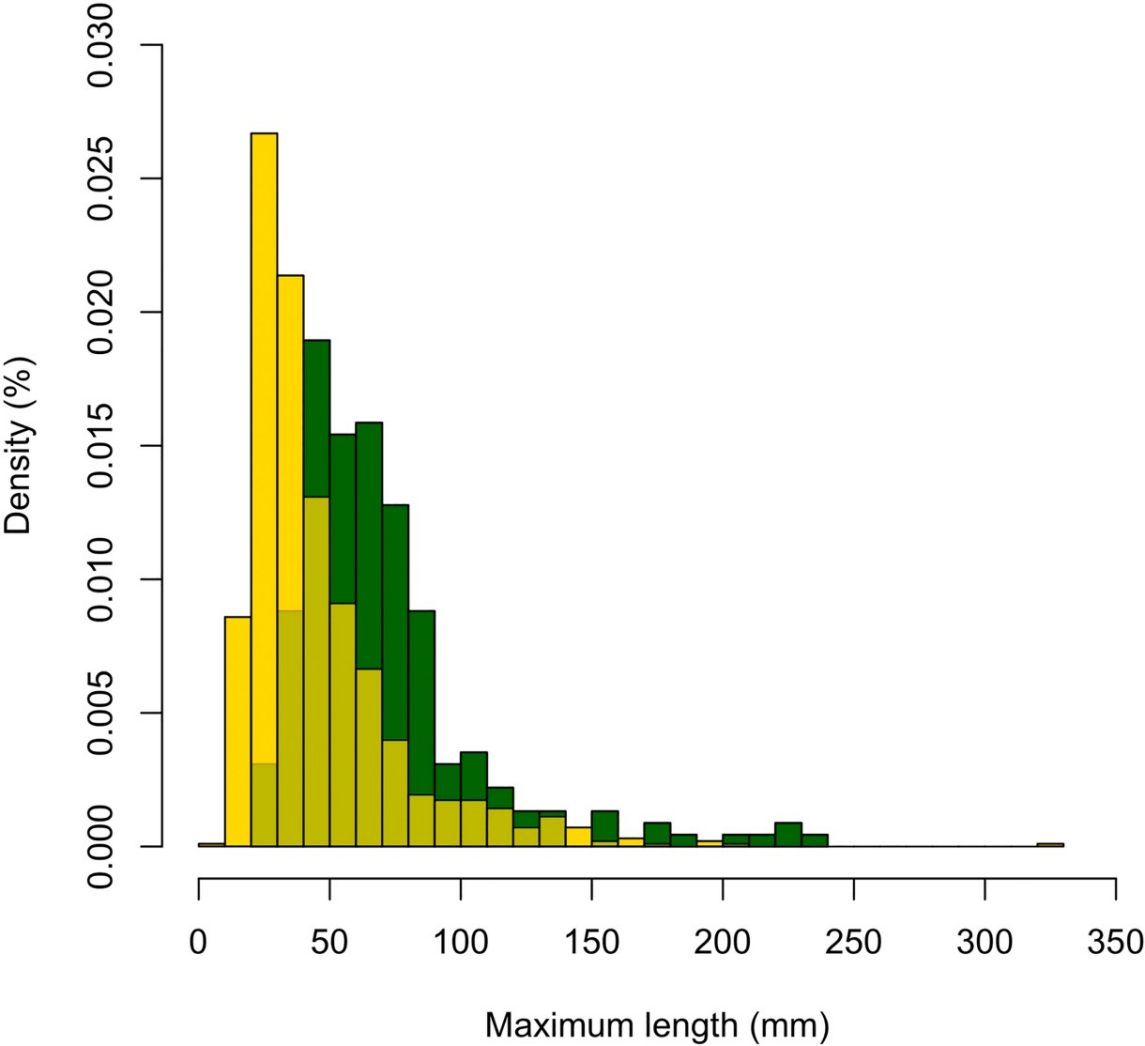
Relative frequencies of faunal fragments from Lingjing, layer 11, per maximum length (mm) size class. Dark green: PBT and RCS combined; Yellow: CCS; Light green: area of overlap between both frequency distributions. Notice the underrepresentation of small faunal remains in the assemblage from the PBT and RCS.

All samples differ significantly from one another when the size of the fragments is considered (Kruskal-Wallis χ^2^ = 174.04, df = 2, *p* < 0.000). This difference is accentuated by the underrepresentation of small fragments in PBT and RCS compared to CCS, which can be explained by the change in recovery procedure of very small fragments implemented in 2017 (Fig 3, Table 2). In all archaeological samples, however, diaphyseal fragments with flake scars are significantly thicker than those without flake scars (Table 4; *t* = -7.3323, df = 166.42, *p* < 0.000), and their cortical thickness indicates most of them comes from medium to large-size mammal long bones. These fragments often also have lengths that nears three times their width.

### Comparison between archaeological and experimental diaphyseal fragments

**Fig 4:**
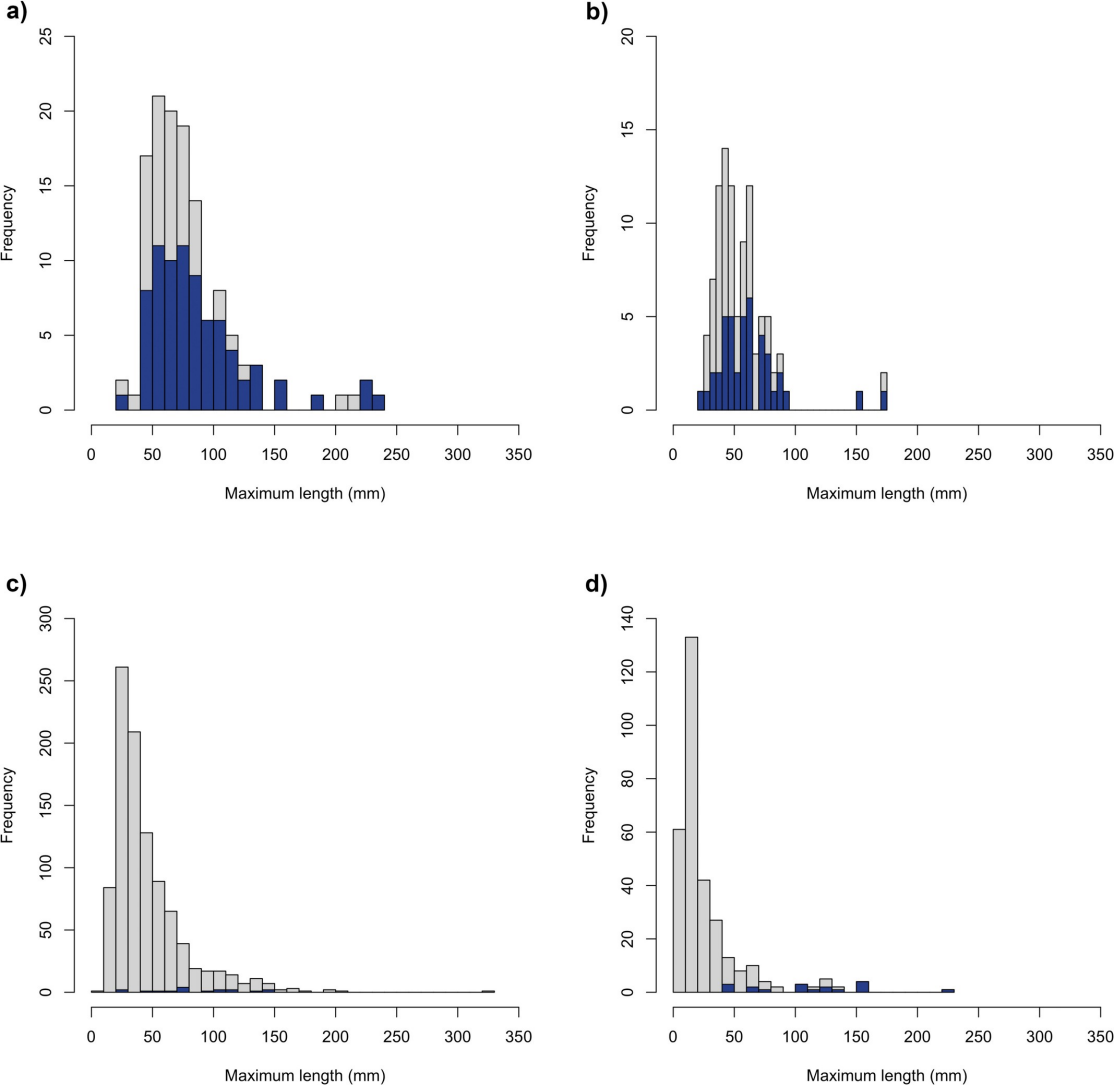
**Frequencies of faunal fragments (in grey) and of specimens bearing flake scars (in blue) per maximum length (mm) size class.** (a) PBT; (b) RCS; (c) CCS; (d) experimental Series 1 and Series 2 combined.

Striking differences appear when comparing archaeological and experimental material. Impact scars and flake scars are systematically associated on our experimental diaphyseal fragments (Table 2). Such an association is rarely observed on the faunal remains from Lingjing. The location and arrangement of flake scars on the experimental fragments show a remarkable similarity with those recorded on the CCS (Table 3). These two sub-samples are also similar in the proportion of faunal remains by size class in general, and the proportion of diaphyseal fragments with flake scars by size class in particular (Fig 4). The specimens from the PBT and RCS samples feature a substantially larger proportion of specimens with bifacial flake scars, respectively 54.5% and 33.3%, than what is observed both on the experimental material and the CCS, respectively 22.2% and 17.6%. The presence of bifacial flake scars on the lateral edges of archaeological specimens is much higher than on their experimental counterpart and 68.6% of the diaphyseal fragments from the PBT and RCS show a pattern of contiguous or interspersed series of flake scars. Such arrangement is extremely rare on the experimental specimens.

**Fig 5:**
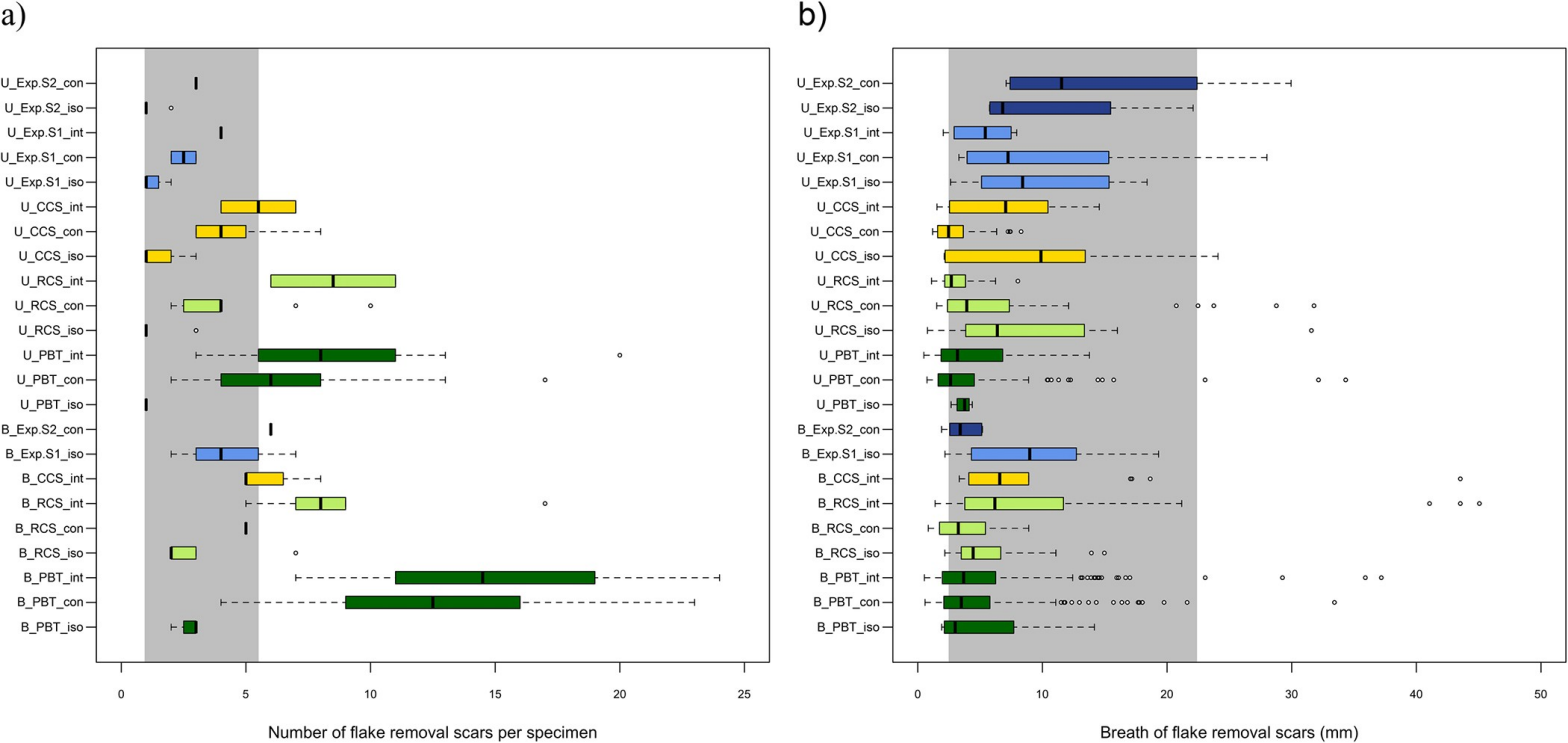
(a) Number, and (b) breadth (mm) of flake scars documented on the specimens considered in the present study by location, arrangement and sub-sample. The sample code contains information on: 1) the location of the flake scars: U = unifacial (no distinction between cortical and medullar surfaces), B = bifacial; 2) the sub-sample: PBT = Potential Bone Tools (dark green), RCS = Restricted Control Sample (light green), CCS = Complete Control Sample (yellow), Exp. S1 = Experimental Series 1 (light blue), Exp. S2 = Experimental Series 2 (dark blue); 3) the arrangement of the flake scars on each specimen: iso = isolated, con = contiguous, int = interspersed series. The grey band refers to μ ± 1σ for the minimal and maximal mean values recorded on the experimental sub-samples.

When the number of the flake scars per specimen is analyzed, significant differences are observed (Fig 5A). These differences are especially marked between PBT and all other samples (*F*_(3,150)_ = 22.78; *p* < 0.000), both archaeological (PBT:RCS *p* < 0.000; PBT:CCS *p* < 0.000) and experimental (PBT:EXP *p* < 0.000). No significant pairwise differences are observed between RCS, CCS and the experimental samples as illustrated by their overlapping values (Fig 5A; RCS:CCS *p* = 0.998; RCS:EXP *p* = 0.796; CCS:EXP *p* = 0.800). Finally, a substantial overlap is also observed for all samples in the breadth of the flake scars regardless of their location or arrangement (Fig 5B).

## Discussion

We argue that a subsample of the remains composing the PBT and RCS from the Lingjing kill/butchery site must be interpreted as expedient bone tools. This diagnosis is based on several lines of evidence. The low percentage of carnivore modifications and the high proportion of remains with cut marks suggest Palaeolithic hominins were the main agent for the accumulation of the faunal assemblage recovered in layer 11 [31, 32, 130, 134, 135]. The uninterrupted vertical distribution of both lithic and animal remains, the low plunge of the lithic artefacts [115], and evidence from lithic refitting [125] argue for a continuous deposition of the archaeological remains between 125 and 105 kyr with minimal post-depositional disturbance. Between 100 and 13.5 kyr, the site appears to have been abandoned, perhaps owing to the drying of the water spring. This change in environmental conditions favoured the accumulation of a ~4.5m loess layer sealing the early late Pleistocene occupation and protecting it from dynamic processes that could have modified the underlying archeological assemblage.

Aware that some peculiar-looking faunal fragments had been isolated during the 2005–2015 excavations owing to their polished surfaces and the presence of flake scars, it was imperative to compare these with a larger sample, i.e., a random selection from the same excavation years and the entire assemblage recovered from layer 11 in 2017. Size difference between the fauna from 2017 and 2005–2015 highlights a bias attributed to the change in recovery methods implemented in 2017, a modification of sampling procedure that had the effect of significantly increasing the proportions of small faunal remains. When both sub-samples from the 2005–2015 excavations were compared, i.e., PBT and RCS, the proportion of fragments with flake scars diminishes from 60.6% in PBT to 44.0% in RCS. Many of these fragments present contiguous, or interspersed series of flake scars. This pattern is even more striking when we consider that only 1.3% of the faunal remains from the 2017 excavations bear flake scars, or 8% when leaving aside the 1,047 remains measuring less than 25mm in length. In the 2017 sample, flake scars are predominantly present on the cortical and medullar surfaces, and at the proximal and/or distal ends of these fragments. It would therefore appear that the difference in excavation methods cannot, in and of itself, explain the differences in the proportion of diaphyseal fragments with flake scars or the location of these scars on the faunal remains.

Mortality patterns, skeletal element representation and anthropogenic modification on the faunal remains [133–136], as well as the osseous and lithic toolkit [31, 32, 124, 125, 130] are coherent with the interpretation according to which Lingjing was repeatedly used as a kill/butchery site during the early Late Pleistocene. In order to explore anthropogenic activities that could have resulted in the production of flake scars on faunal fragments, it became necessary to assess to what extent marrow extraction could generate such a pattern. Our experimental results show that fracturing long bone diaphysis to expose the medullar cavity can produce diaphyseal fragments with flake scars half of the time. However, these scars are found in limited number, rarely exceeding four per fragments, and they seldom occur contiguously or in interspersed series. When both the proportions of faunal fragments in general, and those bearing flake scars in particular, are considered, our experimental data closely matches the pattern emerging from the CCS. Likewise, all the specimens with contiguous flake scars from the RCS fall within the range of variation of our experimental data, both in terms of number of flake scars per item and their breadth. The most important difference between the experimental and the archaeological samples refers to the co-occurrence of impact scars and flake scars. These two anthropogenic modifications were recorded on ~90% of the experimental sample but were seldom observed on archaeological specimens. Finally, we do not find in our experimental material the high prevalence of long bone fragments observed in the PBT sample with numerous contiguous and interspersed series of flake scars. Considering the sedimentary context, the rarity of carnivore modifications on all examined samples and the fact that experimental deliberate flaking of bone fragments of the same type and size produce flake scars comparable to those observed on the archeological specimens [54, 157–159], we must conclude that a subsample of PBT and RCS should be interpreted as composed by bone fragments that were deliberately modified through percussion by Lingjing hominins. The most probable goal of this behaviour was that of using the resulting retouched bone fragments as tools.

The comparison between the archaeological and experimental data suggests a number of qualitative and quantitative criteria could help distinguish faunal remains with flake scars that were intentionally modified for technological purpose from those that result from carcass processing activities such as marrow exploitation, even in the absence of a well-developed use wear polish. From a contextual perspective, if carnivores had a limited role in the accumulation, or attrition [e.g., 160], of the faunal assemblage and if this assemblage was not subjected to important dynamics processes after its deposition, a low percentage in the co-occurrence of marrow extraction impact scars and flake removal scars on diaphyseal fragments from medium to large-sized mammal long bones is a good indicator that some of these specimens may have been intentionally shaped by direct percussion. From a quantitative perspective, this interpretation can be further supported when specimens bear more than six flake scars and when their arrangement show a high frequency of contiguous, and/or interspersed series of, scars. Although most of the fragments in our experimental sample bore four scars or less, we err on the side of caution and extend this threshold to include values included between μ and μ + 1σ. Our conclusions are almost entirely compatible with those reached by Backwell and d’Errico [7]. Their comparison between a large sample from Olduvai and an experimental sample knapped on elephant bones led these authors to suggest that diaphyseal fragments “bearing five or more flake scars, some of which are contiguous, with one or more anomalously invasive [i.e., larger than 40mm in breadth] primary removals” [7] were likely to have been intentionally modified into expedient tools. In our analysis, however, the breadth of flake scars doesn’t seem to be a good indicator for the intentional shaping of diaphyseal fragments. This may be due to the fact that Backwell and d’Errico experimented on elephant bones, which allows the development of more invasive flake scars. Simply put, flake scars size doesn’t seem to matter as much as their frequency and arrangement.

**Fig 6:**
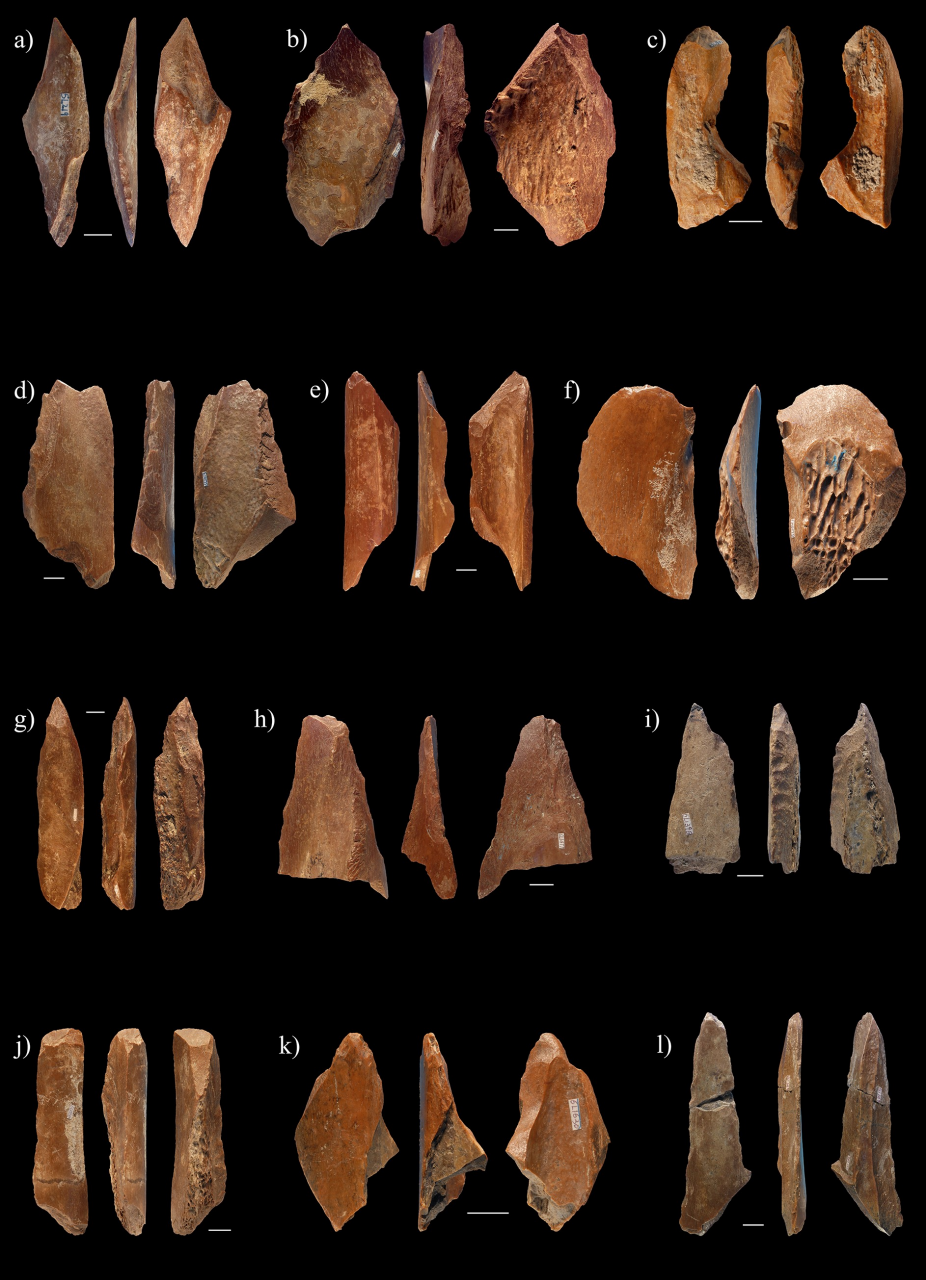
Sample of diaphyseal fragments bearing flake scars from Lingjing, layer 11, interpreted as expedient osseous tools. Refer to S1 Table for data. Scales = 1cm.

**Fig 7:**
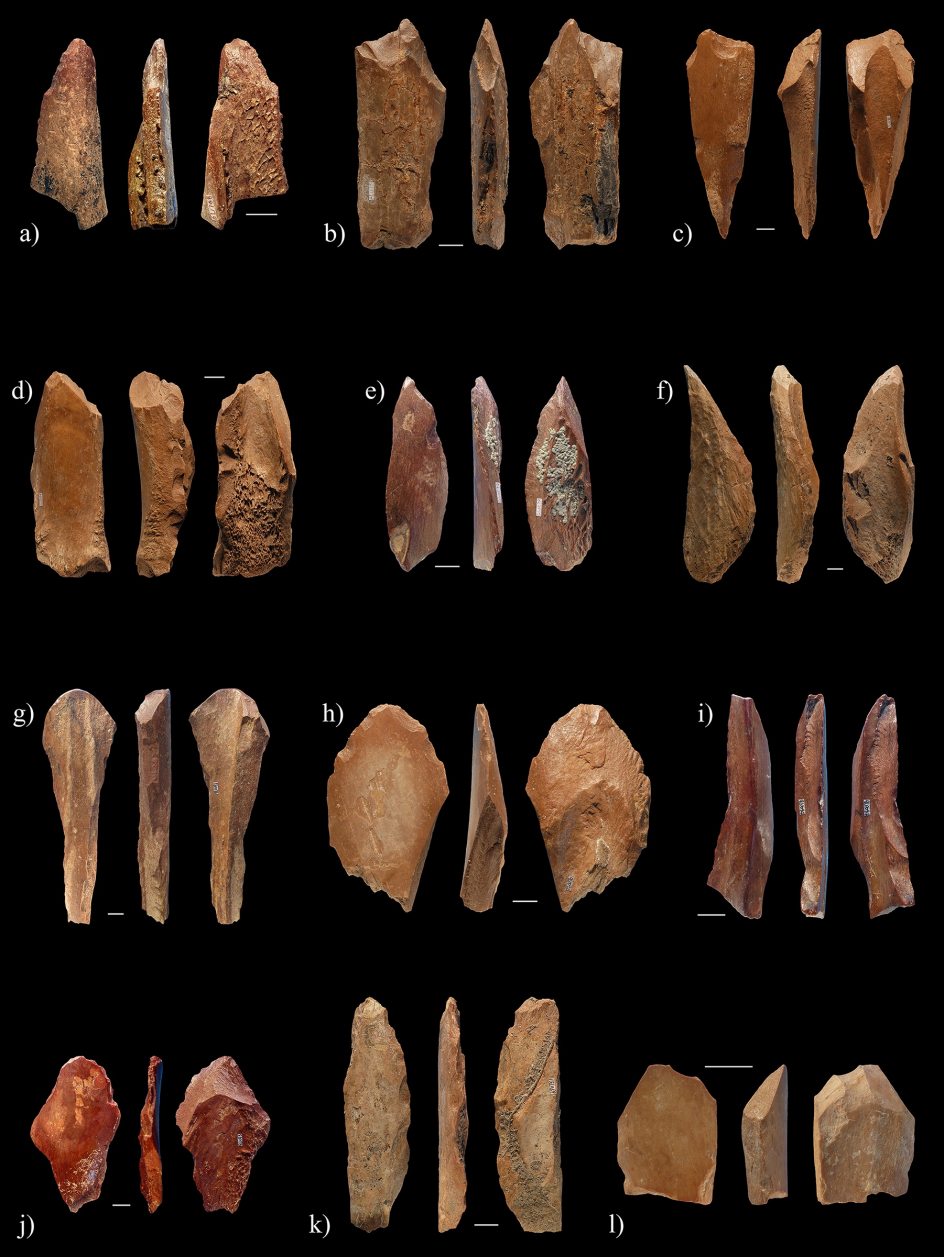
Sample of diaphyseal fragments bearing flake scars from Lingjing, layer 11, interpreted as expedient osseous tools. Refer to S1 Table for data. Scales = 1cm.

**Fig 8:**
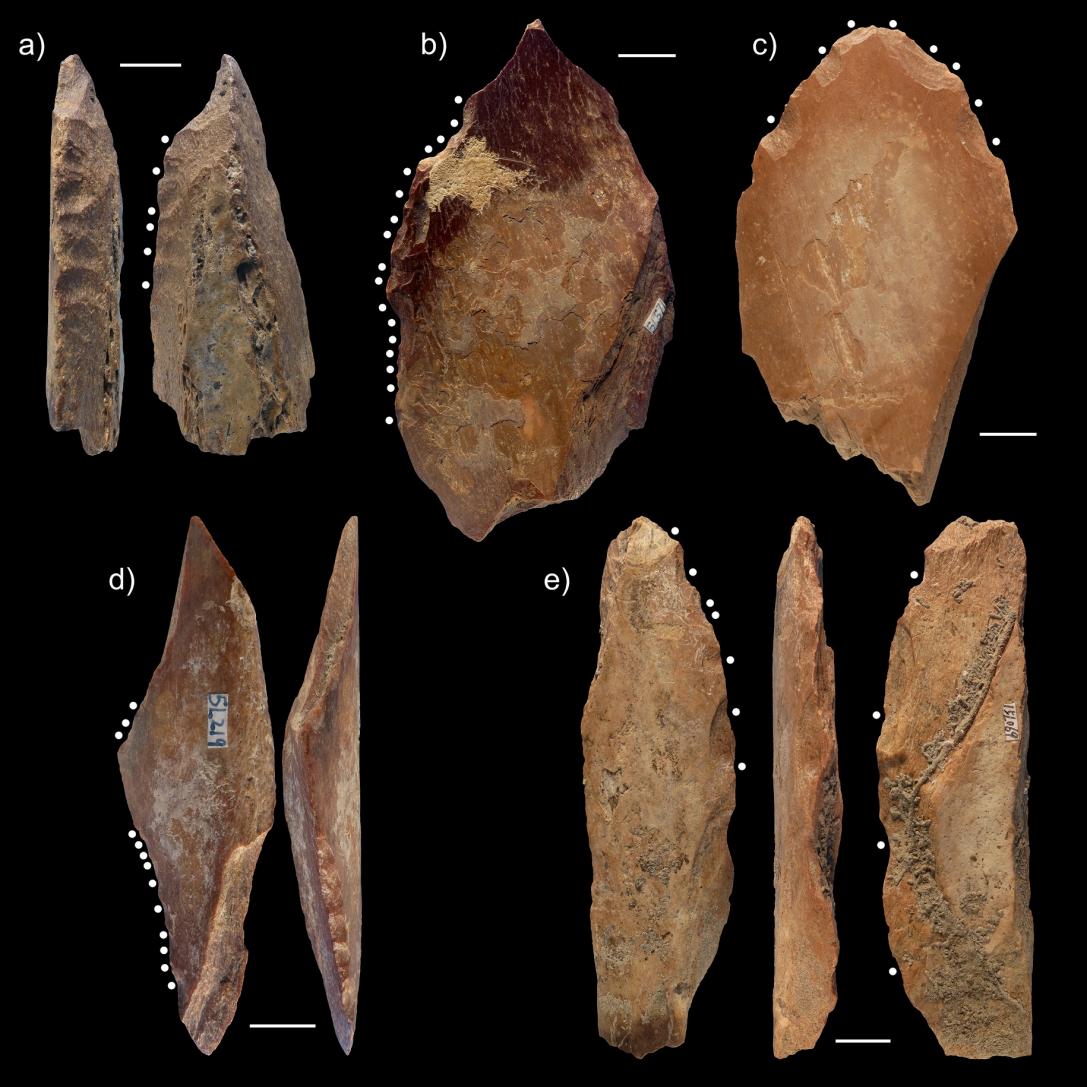
Close-up views of a sample of diaphyseal fragments bearing flake scars from Lingjing, layer 11. Dots indicate the location of flake scars produced by direct percussion. Notice the variability in the flaking pattern and distribution. Scales = 1cm.

Based on these criteria, we can interpret 56 diaphyseal fragments, i.e., 49 of the PBT and 7 in the RCS, from the Lingjing, layer 11, faunal assemblage considered in the present study as having been intentionally modified by direct percussion (Figs 6 and 7, S1 Table). Their compact bone thicknesses measure 14.6mm on average (σ = 5.51mm), and their maximum lengths are usually 2.72 times longer than their widths (σ = 0.87). More than three quarters of them (78.6%) show evidence of fresh fractures which suggests they were modified while the bone was still green. Two thirds of them (67.9%) bear flake scars on both their cortical and medullar surfaces. The number of flake scars per specimens varies from seven to 24 (μ = 12.8; σ = 5). The lateral edges of the diaphyseal fragments are often modified (cortical surface: 71.4%; medullar surface = 42.9%) with either contiguous (51.8%) or interspersed series (48.2%) of flake removals that regularly extend up to the proximal or distal end of the fragments (Fig 8).

**Fig 9:**
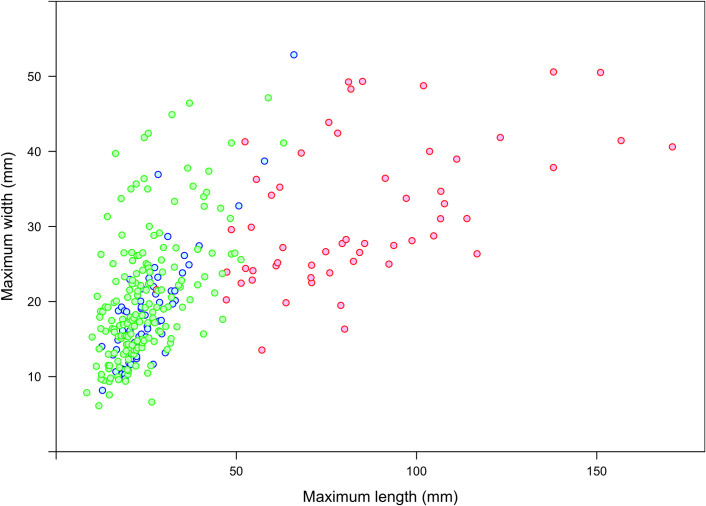
**Morphometric comparison between the unretouched lithic flakes (green), lithic tools (blue) and expedient osseous tools (red) from Lingjing, layer 11.** Data for the lithic remains extracted from [125, Fig 3].

The production of expedient bone tools at Lingjing provides a new outlook on the prehistoric lifeways of the human groups who visited the site. The presence of a water spring in a grassland-dominated environment with a mosaic of scattered, mixed forests surely attracted both animals and humans, and provided these individuals with a reliable hunting spot at the beginning of the Late Pleistocene. When undertaking a hunting trip, these hunters could anticipate their needs at the hunting grounds and collect a few quartz and quartzite pebbles along the way in the riverbeds located in the vicinity of the site to complement the few tools made of allochthonous material they had in their possession. Following a successful kill, lithic tool manufacture and butchery activities appear to have been undertaken at the site. Although some steps of the operational sequence guiding the production of expedient bone tools are still missing, it appears the Lingjing visitors targeted thick, elongated diaphyseal fragments to modify their edges by direct percussion. The fractures present on these tools indicate bone fragments were knapped while still being fresh. A thorough survey of the location of impact scars on medium-sized mammalian long bones could help us determine whether or not a particular fracturing method was implemented in order to access the marrow while producing elongated diaphyseal fragments. The predominance of flake removal scars on lateral edges, sometime extending all the way to the proximal or distal end of the fragment, implies a modification oriented towards the production of a long, sharp edge. Comparing the size of these fragments with that of unretouched lithic flakes and lithic tools from the same layer reveals an interesting pattern (Fig 9). Such comparison suggests these bone fragments may have been specifically targeted by the humans visiting Lingjing to complement the small size of the lithics composing their toolkit. The function of the bone tools is a topic to be explored. However, considering that processing carcasses of large and medium size prey has certainly been one of the functions the site has fulfilled, it is likely that these expedient tools were used in butchery or hide processing activities. An experimental and use wear program is currently being implemented to test this hypothesis.

Our results have implications on our understanding of human behavioural variability during the Middle to Late Pleistocene transition in China. Research undertaken at Lingjing shows the importance of bone as a raw material in the technological system of the human groups that visited the site during this period. Bone tools were used in a variety of stone knapping activities [31, 32], as implements fit to knap bones to allow marrow extraction [130], and as a mean to permanently record information in the form of engraved patterns [143]. When adding the evidence from the present study, it appears clearly that the visitors at Lingjing not only understood the mechanical properties of osseous raw material but, most importantly, knew how to take advantage of them in a variety of subsistence, and perhaps symbolic, activities. The diversity of functions for which bone tools were used is also compelling. It reinforces the view that the technological system at Lingjing likely represents an expression of a long-lasting tradition whose origin and development remain to be established [32]. On the other hand, the Lingjing case further highlights the inability of lithic technology to adequately describe the whole breadth of behavioural variability for the humanities that preceded us. Careful consideration of the faunal assemblages, both from a taphonomic and a technological perspective, especially in East Asia, now allow us to perceive a level of technological complexity that is entirely comparable to penecontemporaneous evidence from other regions of the Old World [23, 116, 143, 161–165].We can only hope the recent discoveries from Lingjing and other sites will encourage a careful re-examination of faunal assemblages from these perspectives to further our understanding of the cultural trajectories of the technological systems before and after the dispersal of our species in the region.

## Supporting information

S1 Table Contextual, taphonomic, and morphometric data for the diaphyseal fragments bearing flake scars from Lingjing, layer 11, and interpreted as expedient osseous tools. *Note*: With regards to the breadth of flake scars, empty cells were added to signal a discontinuity between two flake scars or series of flake scars. This affects only the specimens on which interspersed series were observed.
(XLSX)
